# Whey Protein–Polysaccharide Nanocomplexes: Structure–Function Characterization and Bone-Related Outcomes in Ovariectomized Rats

**DOI:** 10.3390/nu18203321

**Published:** 2026-10-10

**Authors:** Juana Isela Rojas-Molina, Elsa Gutiérrez-Cortez, Ricardo Moisés González-Reza, Omar M. Gomez-Vazquez, Brandon Correa-Piña, Ezequiel Hernández-Becerra, Daniel Jiménez-Mendoza, Alejandra Rojas-Molina, Carlos Quirino-Barreda, Michelle Quintero-García

**Affiliations:** 1 Laboratorio de Investigación Química y Farmacológica de Productos Naturales, Facultad de Química, Universidad Autónoma de Querétaro, Cerro de las Campanas S/N, Santiago de Querétaro C.P. 76017, Querétaro, Mexico; rojasa@uaq.mx; 2 Laboratorio de Procesos en Ingeniería Agroalimentaria, Unidad de Investigación Multidisciplinaria (UIM), FES-Cuautitlán, Universidad Nacional Autónoma de México, Cuautitlán Izcalli C.P. 54714, State of Mexico, Mexico; elsane@comunidad.unam.mx (E.G.-C.); gonzalez.reza@comunidad.unam.mx (R.M.G.-R.); 3 Centro de Física Aplicada y Tecnología Avanzada, Universidad Nacional Autónoma de México, Campus Juriquilla, Santiago de Querétaro C.P. 76230, Querétaro, Mexico; omar_govaz@hotmail.com (O.M.G.-V.); BrACorreap@outlook.com (B.C.-P.); 4 Escuela de Bachilleres, Plantel Norte, Universidad Autónoma de Querétaro, Av Cerro Sombrerete, Desarrollo San Pablo S/N, Santiago de Querétaro C.P. 76125, Querétaro, Mexico; ezequiel.hernandez@uaq.edu.mx; 5 Departamento de Ingeniería Física, División de Ciencias e Ingenierías, Universidad de Guanajuato, Campus León, Lomas del Bosque 103, Col. Lomas del Campestre, León C.P. 37150, Guanajuato, Mexico; jmd_pepe@yahoo.com.mx; 6 Departamento de Ingeniería Electromecánica, Tecnológico Nacional de México/ITS de Purísima del Rincón, Blvd. Del Valle 2301, Col. Guardarrayas, Purísima del Rincón C.P. 36413, Guanajuato, Mexico; 7 División de Ciencias Biológicas y de La Salud, Departamento de Sistemas Biológicos, Unidad Xochimilco, Universidad Autónoma Metropolitana, Mexico City C.P. 04960, Mexico; cquirino@correo.xoc.uam.mx; 8 Departamento de Neurobiología Celular y Molecular, Instituto de Neurobiología, Universidad Nacional Autónoma de México, Campus Juriquilla, Santiago de Querétaro C.P. 76230, Querétaro, Mexico; adr_mich@hotmail.com

**Keywords:** protein–polysaccharide nanocomplexes, whey protein, *Opuntia ficus-indica* mucilage, *Tamarindus indica* xyloglucan, ovariectomy, bone mineral density, bone microarchitecture, hydroxyapatite

## Abstract

**Background/Objectives:** Estrogen deficiency is associated with impaired bone mineralization and deterioration of bone quality. Protein–polysaccharide nanostructures have attracted interest as food-grade formulations because their physicochemical properties can be modified by the nature and organization of their constituent biopolymers. This study evaluated the effects of whey protein–polysaccharide nanocomplex formulations on bone-related outcomes in ovariectomized female Wistar rats. **Methods:** Whey protein nanocomplexes containing either *Opuntia ficus-indica* mucilage or *Tamarindus indica* xyloglucan were produced by complex coacervation and ultrasonication. The nanocomplexes were characterized in terms of particle size, polydispersity index, ζ-potential, and morphology. Ovariectomized rats received the nanostructured formulations, their corresponding physical mixtures, the individual ingredients, or control diets for 12 weeks. Femoral bone quality was evaluated through biomechanical testing, calcium and phosphorus content, Ca/P ratio, bone mineral density, scanning electron microscopy and histomorphometry, and X-ray diffraction analysis. **Results:** The developed nanocomplexes exhibited particle sizes below 500 nm and polydispersity indices of approximately 0.3. Their physicochemical characteristics and morphology differed according to the polysaccharide incorporated. Ovariectomy was associated with unfavorable changes in bone mineralization, microarchitecture, crystallographic characteristics, and biomechanical competence. Among the evaluated treatments, the whey protein–*O. ficus-indica* mucilage nanostructured formulation (LMCC) showed the most favorable overall pattern across most bone-related outcomes, including bone mineral density, femoral calcium content, microarchitecture, hydroxyapatite crystallite size, and biomechanical properties. However, the response to nanostructuring was not uniform across the two polysaccharide systems, and the xyloglucan-based formulations exhibited parameter-dependent responses. **Conclusions:** The biological response to whey protein–polysaccharide formulations was associated with polysaccharide type and physicochemical organization. The mucilage-based nanostructured formulation showed a more favorable overall pattern across most evaluated bone-related outcomes in ovariectomized rats. However, calcium association within the nanostructures, calcium-release kinetics, gastrointestinal bioaccessibility, and intestinal calcium absorption were not evaluated; therefore, the mechanisms underlying these responses remain to be established. These findings should be interpreted as preclinical and should not be extrapolated directly to osteoporosis prevention in humans.

## 1. Introduction

Calcium is an essential mineral in the human body and represents the fifth most abundant element in the organism. Approximately 99% of total body calcium is localized within the skeletal system in the form of hydroxyapatite [1]. Skeletal calcium plays a dual role: it provides structural strength to bones and serves as a dynamic reservoir for maintaining serum calcium homeostasis [2].

Menopause is defined as the permanent cessation of menstruation, diagnosed retrospectively after 12 consecutive months of amenorrhea due to ovarian follicle atresia and a reduced capacity to produce estrogen. The most characteristic symptoms associated with menopause include vasomotor disturbances (e.g., hot flashes), osteoporosis, and an increased risk of cardiovascular diseases, among others [3]. Osteoporosis, in particular, is a chronic metabolic bone disease characterized by decreased bone mass and deterioration of bone microarchitecture, leading to increased bone fragility and susceptibility to fractures [4].

Current therapeutic approaches for osteoporosis prevention and management often involve calcium supplementation combined with vitamin D as adjuvants. In addition, several FDA-approved pharmacological agents, such as bisphosphonates, calcitonin, estrogen therapy, and parathyroid hormone, are available. However, their use has been associated with numerous adverse effects, including atypical femoral fractures, increased incidence of coronary artery disease, stroke, thromboembolic events, breast cancer, and gallstones [5]. Similarly, mineral supplements may cause drug interactions and gastrointestinal discomfort, such as constipation, flatulence, and dyspepsia, and may also increase the risk of kidney stone formation. Moreover, the fraction of calcium supplied by conventional supplements that is ultimately absorbed by the body remains relatively low [6].

Recent advances have emphasized the potential of natural polymers, or biopolymers, as promising materials for the development of functional food formulations, particularly in the form of micro- and nanoparticles. Their attractiveness stems from their wide availability, low cost, biodegradability, biocompatibility, and versatility for the incorporation and stabilization of bioactive compounds [7]. Micro- and nanoparticles may also modify the physicochemical environment of incorporated compounds and influence their stability and functional performance [8].

Biopolymers such as mucilage extracted from *Opuntia ficus-indica* cladodes and xyloglucan derived from *Tamarindus indica* seeds represent a novel and underexplored strategy for the synthesis of micro- and nanoparticles with potential for calcium supplementation. Our research group has previously characterized the physicochemical properties of these biopolymers and demonstrated their suitability for the formation of micro- and nanospheres [9,10]. Moreover, the bioavailability of calcium derived from mucilage has been investigated in both growing and osteoporotic rat models, yielding promising results [11,12].

An important property of these polysaccharides is their resistance to enzymatic digestion in the upper gastrointestinal tract. In the colon, however, they are fermented by the gut microbiota, producing short-chain fatty acids (SCFAs). SCFAs have been shown to enhance mineral absorption, including calcium, and to promote bone health by inhibiting osteoclastogenesis and reducing bone resorption [13,14].

Furthermore, whey protein is a food-grade ingredient with nutritional value and functional properties that make it suitable for incorporation into protein–polysaccharide formulations. As a by-product of the dairy industry, whey protein represents a sustainable and accessible ingredient for the development of functional food systems [15,16].

One of the main advantages of employing biodegradable biopolymers in the development of micro- and nanostructures is their abundance in nature, including their occurrence in industrial by-products, as well as their inherent biocompatibility, flexibility, non-toxicity, low cost, and renewable origin [17]. Protein–polysaccharide micro- and nanostructures have been widely investigated as food-grade systems for the incorporation and stabilization of bioactive compounds. Depending on their composition and preparation method, these systems may modify the physicochemical environment of incorporated compounds and influence their stability and functional performance [18,19].

Nano- and submicron-scale systems are widely investigated for the incorporation and stabilization of bioactive compounds and nutrients. Depending on their composition, preparation method, and intended application, these systems may span a broad particle-size range and can entrap, adsorb, associate, or otherwise interact with functional compounds within a polymeric matrix. Their reduced dimensions and high surface-area-to-volume ratio may influence the physicochemical stability and functional properties of incorporated compounds [20,21].

Moreover, sonication is widely recognized as an efficient method for nanoparticle synthesis and dispersion, as it facilitates the disintegration of agglomerates and promotes the formation of uniformly sized particles through the high-energy effects generated by acoustic cavitation [22]. Although protein–polysaccharide complexes have been extensively investigated as carriers or structured matrices for bioactive compounds, information regarding their effects on bone-related outcomes remains limited. Furthermore, few studies have directly compared the biological performance of free polysaccharides, physical mixtures, and nanostructured complexes obtained by complex coacervation in vivo [23].

The objective of this study was to evaluate the effects of whey protein–polysaccharide nanocomplex formulations on bone quality in ovariectomized rats as an experimental model of estrogen-deficiency-associated bone loss. We hypothesized that nanostructuring of whey protein–polysaccharide complexes through complex coacervation and ultrasonication could modify the biological response relative to the corresponding physical mixtures and individual ingredients, as reflected in bone mineral density, biomechanical properties, mineral content, microarchitecture, and crystallographic characteristics.

## 2. Materials and Methods

### 2.1. Vegetal Material

Mucilage was obtained from *Opuntia ficus-indica* cladodes harvested during the spring of 2024 and collected at 135 days of maturity stage from the sprout in the experimental field of Amazcala in the Engineering Department of the Autonomous University of Querétaro. The xyloglucan was extracted from *Tamarindus indica* seeds provided by PRODIASA, S.A. de C.V. Mucilage and xyloglucan extraction was carried out according to the methodology reported by Quintero-García et al. [9] and Espíndola-Sotres et al. [10], respectively.

### 2.2. Experimental Animals

All experimental procedures were performed in compliance with the guidelines established by the Mexican Official Standard NOM-062-ZOO-1999 [24], Technical Specifications for the Production, Care, and Use of Laboratory Animals. The study protocol was registered with the Research and Postgraduate Office of the Autonomous University of Querétaro (FQU202419) and approved by the Institutional Ethics Committee under the authorization code FONFIVE-CEI-64/24. Female Wistar rats (two months old, weighing 200–250 g) that had undergone bilateral ovariectomy were supplied by BIOINVERT (Integrated Biomedicine for Vertebrates). Animals with a body weight below 200 g were excluded from the surgical procedure to minimize the risk of perioperative complications. Following surgery, the animals were housed in standard cages under controlled temperature conditions (22 °C) with a 12:12 h light–dark cycle for a two-week acclimatization period. Food and water were available ad libitum throughout the study. All procedures related to waste management were conducted in accordance with the Mexican Official Standard NOM-087-SEMARNAT-SSA1-2002 [25]. Throughout the study, animals were inspected daily by trained personnel to evaluate general health condition, food and water consumption, mobility, grooming behavior, and the presence of any signs of pain, distress, or postoperative complications. Humane endpoints were established prior to study initiation. Animals showing severe weight loss (>20% of initial body weight), persistent lethargy, inability to access food or water, severe infection, or signs of unrelieved pain or distress were to be removed from the study and humanely euthanized. No animals met these criteria during the experimental period.

### 2.3. Development of Whey–Xyloglucan and Whey–Mucilage Protein Nanocomplexes by Complex Coacervation

The nanocomplexes were prepared according to the methods reported by González-Martínez et al. [26] and Otálora et al. [27], with modifications. A 2:1 whey protein-to-polysaccharide ratio was used for both formulations, namely whey–xyloglucan and whey–mucilage. All dispersions were maintained under constant stirring at 350 rpm.

The whey protein dispersion was adjusted to pH 2.0 using 0.1 M HCl, whereas the xyloglucan and mucilage dispersions (0.0625% *w*/*w*) were separately adjusted to pH 13.0 using 0.1 M NaOH. The whey protein dispersion was then added dropwise to each polysaccharide dispersion separately under continuous stirring.

The resulting protein–polysaccharide systems were subjected to ultrasonication for 5 min at 60 W using an ultrasonic processor (Hielscher UP400St, Teltow, Germany) equipped with an H22L2D sonotrode with a 22 mm tip diameter, operating at 24 kHz in continuous mode. Following ultrasonication, the pH of each nanostructured system was adjusted to 3.0. The resulting suspensions were stored at 4 °C until further analysis.

#### 2.3.1. Nanocomplex Characterization

Particle size (PS), polydispersity index (PDI), and ζ-potential of the nanocomplexes were determined using a particle size analyzer (ZetaSizer Pro, Malvern Instruments, Worcestershire, UK). Prior to analysis, all samples were appropriately diluted with Milli-Q water (Millipore, Milli-Q Direct 8, Bay City, MI, USA) to achieve optimal scattering intensity. Measurements were performed at 25 °C using a disposable DTS1070 capillary cell (Malvern Panalytical, Westborough, MA, USA). Particle size values were calculated as the mean of five independent measurements. The ζ potential was determined by electrophoretic light scattering, and the electrophoretic mobility was converted to ζ potential using the Helmholtz-Smoluchowski equation [28]. Data acquisition and analysis were performed using ZS Xplorer software version 2.01 (Malvern Panalytical, Westborough, MA, USA).

#### 2.3.2. Electron Microscopy Analysis

Electron microscopy was used to evaluate the morphology of the whey protein–polysaccharide nanocomplexes formulated with *Opuntia ficus-indica* mucilage and *Tamarindus indica* xyloglucan. Representative micrographs were acquired using a Hitachi SU8230 field-emission scanning electron microscope (Hitachi High-Technologies Corporation, Tokyo, Japan) at an accelerating voltage of 30.0 kV and a working distance of 7.9 mm.

The whey protein–mucilage nanocomplexes were imaged in bright-field scanning transmission electron microscopy (BF-STEM) mode at ×100,000 magnification, whereas the whey protein–xyloglucan nanocomplexes were imaged using secondary-electron imaging [SE(UL)] at ×200,000 magnification. The corresponding scale bars were 500 nm and 200 nm, respectively.

The resulting micrographs were used to evaluate particle morphology, degree of aggregation, and overall structural organization of the nanocomplexes. Morphological observations were subsequently compared with particle-size distribution, polydispersity index, and ζ-potential measurements.

### 2.4. Experimental Design

All animals, except those assigned to the Sham group, underwent bilateral ovariectomy. Following the acclimatization period, animals were randomly allocated to the experimental groups using an electronic random number generator (GraphPad Prism 7.02, GraphPad Software Inc., San Diego, CA, USA). No animals were excluded after randomization, and all animals completed the study. A total of 80 animals were housed individually in stainless-steel metabolic cages under controlled environmental conditions (22 °C and a 12 h:12 h light–dark cycle). The experimental groups consisted of eight animals per group. The individual animal was considered the experimental unit. The number of animals per group was selected based on previous studies using similar experimental models and outcome variables [29]. The experimental groups were as follows: Ctrl, ovariectomized rats fed the standard AIN-93M maintenance diet; CCa, ovariectomized rats fed the AIN-93M diet with calcium adjusted using calcium citrate; LXMix, ovariectomized rats fed the AIN-93M diet supplemented with a whey–xyloglucan mixture in the same proportion used for nanosphere preparation; LXCC, ovariectomized rats fed the AIN-93M diet supplemented with whey–xyloglucan nanospheres obtained by complex coacervation; XLG, ovariectomized rats fed the AIN-93M diet supplemented with xyloglucan; Whey, ovariectomized rats fed the AIN-93M diet supplemented with whey protein concentrate (CYTECSA, Mexico City, Mexico); LMMix, ovariectomized rats fed the AIN-93M diet supplemented with a whey–mucilage mixture in the same proportion used for nanosphere preparation; LMCC, ovariectomized rats fed the AIN-93M diet supplemented with whey–mucilage nanospheres obtained by complex coacervation; MOF, ovariectomized rats fed the AIN-93M diet supplemented with mucilage; and Sham, non-ovariectomized rats fed the standard AIN-93M diet.

All experimental diets were formulated according to the AIN-93M recommendations. The detailed composition and calculated nutritional content of the experimental diets are presented in Table 1. All diets included a vitamin mixture (AIN-93-VX; Harlan Inc., Indianapolis, IN, USA; TD 94047) and a calcium-free mineral mixture (AIN-93-MX; Harlan Inc., Indianapolis, IN, USA; TD 04374). Dietary calcium was formulated to provide approximately 5 g/kg across all experimental diets. When the calculated calcium contribution from mucilage, xyloglucan, or whey protein concentrate was insufficient to reach the target dietary calcium level, calcium carbonate (CaCO_3_; Merck 2066, Darmstadt, Germany) was added as a supplemental calcium source. In the Ctrl and Sham diets, CaCO_3_ was used as the dietary calcium source.

All experimental diets were formulated according to AIN-93M recommendations and were designed to provide comparable energy and macronutrient profiles. Calculated calcium content was approximately 5 g/kg in all diets, with only minor variation among formulations. Protein, carbohydrate, lipid, and energy contributions were also closely comparable across groups. However, the amounts of the experimental ingredients (whey protein, mucilage, xyloglucan, and the corresponding mixtures or nanostructured formulations) were not dose-matched among all treatment groups, because some ingredients also contributed to meeting the overall nutritional composition of the diets.

The experimental period lasted 12 weeks. During this time, animals received their respective diets and water ad libitum, and body weight was recorded weekly. Food intake was monitored during the experimental period using the available feeding records. For each recorded interval, food consumption was expressed as the estimated amount consumed per rat per day, taking into account the number of animals represented in each group and the duration of the interval. Estimated daily calcium intake was calculated by multiplying food intake (g/rat/day) by the formulated dietary calcium concentration (5 mg/g). Thus, calcium intake was estimated from the dietary formulation and was not determined by direct chemical analysis of the consumed feed. Group allocation was known to personnel involved in animal care and dietary administration. During BMD assessment, the researcher responsible for maintaining the experimental records was aware of group allocation, whereas radiographic image analysis and BMD calculation were performed by a second researcher blinded to treatment allocation. Femoral samples were coded before biomechanical and microscopic analyses to conceal treatment allocation from the personnel performing these assessments. At the end of the experimental period, animals were euthanized by gradual-fill carbon dioxide (CO_2_) exposure followed by confirmation of death, in accordance with the recommendations of NOM-062-ZOO-1999 [24].

### 2.5. Analysis of Serum Estrogen Levels

To verify the effectiveness of ovariectomy, blood samples were collected from the caudal vein one day before treatment initiation and at the end of the experimental period. Serum was separated by centrifugation at 3000 rpm (LXJ-802, Thermo Scientific, Pittsburgh, PA, USA). Estrogen concentrations were determined using a commercial ELISA kit (Cat. No. 582251-96S, Cayman Chemical Company, Ann Arbor, MI, USA) according to the manufacturer’s instructions and the procedure described by Quintero-García et al. [12].

### 2.6. Assessment of Femoral Weight, Dimensions, and Biomechanical Properties

Experimental animals were fasted for 12 h prior to euthanasia. Subsequently, the right and left femurs were excised, and all adherent soft tissues and bone marrow were carefully removed. Femoral weight, length, width, and thickness were recorded according to a previously described protocol [31].

Following collection, femurs were wrapped in saline-moistened gauze and stored at −20 °C until analysis. Prior to biomechanical testing, samples were allowed to equilibrate to room temperature.

Femoral biomechanical properties were evaluated using compression and three-point bending tests performed on a universal testing machine (Zwick/Roell, Ulm, Germany; Model Z005, load cell 5000 N) equipped with TestXpert Intelligent Testing software (version 12.0). Maximum compressive force (Fmax, N) and maximum load at failure during three-point bending (Pmax, N) were recorded as indicators of femoral mechanical strength. All measurements were performed according to the manufacturer’s instructions and previously reported methodologies [12,31].

### 2.7. Determination of Calcium and Phosphorus Content in the Femurs

Calcium and phosphorus contents in left femoral bone samples were determined by inductively coupled plasma optical emission spectrometry (ICP-OES; Agilent 730-ES, Santa Clara, CA, USA), following nitric acid digestion according to previously reported methodologies [12,31].

### 2.8. Determination of Microarchitecture of Rat Femoral Bone

Microstructural parameters of the femoral bone, including trabecular separation (Tb.Sp), trabecular thickness (Tb.Th), and cortical thickness (Ct.Th), were evaluated using a scanning electron microscope (JEOL JSM-6060LV, Akishima, Tokyo, Japan) according to previously described methods [11,12,31].

### 2.9. Analyses of Bone Mineral Density

Rats from all experimental groups underwent densitometric analysis at the beginning and at the end of the study to determine bone mineral density (BMD). Measurements were performed using a conventional X-ray system (Satelec X-mind^®^, Cologne, France) operating at 70 kV and 8 mA, with a wavelength of 0.177 × 10^−10^ m (Toshiba X-ray tube DG-073B-DC, Tokyo, Japan), coupled to a detector (Handy Medical Equipment HDR-460 sensor, Shanghai, China) and a dedicated computer platform [31,32].

The system was calibrated during each measurement session using the same bovine femoral cortical bone standards of different thicknesses described by Jiménez-Mendoza et al. [32]. These standards, whose density had been determined gravimetrically, were used to establish the relationship between X-ray attenuation and bone mineral density according to the previously published single-energy X-ray absorptiometry methodology. Radiographic images were acquired using the software supplied with the detector, whereas BMD was calculated using the software developed by Jiménez-Mendoza et al. [32] for processing radiographic image data. BMD values were expressed as g/cm^2^.

### 2.10. Assessment of Femoral Microstructural Properties by X-Ray Diffraction

X-ray diffraction (XRD) analysis was performed to evaluate the crystalline characteristics of femoral bone mineral. Femoral bone samples were cleaned of adhering soft tissues, dried, and finely ground to obtain a homogeneous powder. The resulting powders were analyzed using an XRDynamix 500 diffractometer (Anton Paar GmbH, Graz, Austria) equipped with Cu Kα radiation (λ = 1.5406 Å). Diffraction patterns were collected under conditions suitable for identifying the principal reflections of biological hydroxyapatite. The diffraction peaks were indexed according to the hydroxyapatite crystallographic structure. Diffraction data were processed using OriginPro 2025 (OriginLab Corporation, Northampton, MA, USA).

Background subtraction and peak-profile fitting were performed prior to peak-width analysis. The hydroxyapatite (002) reflection was selected for crystallite-size determination. The full width at half maximum (FWHM) was obtained from the fitted (002) peak as the diffraction-profile width measured at 50% of its maximum intensity after background subtraction. To account for instrumental broadening, a lanthanum hexaboride (LaB_6_) standard was measured under the same instrumental conditions used for the bone samples. Assuming Gaussian peak broadening, the corrected peak width (*β*) was calculated according to

$$\beta =\sqrt{\beta_{\mathrm{measured}}^{2}-\beta_{\mathrm{instrumental}}^{2}}$$

where $\beta_{\mathrm{measured}}^{2}$ is the experimental FWHM of the hydroxyapatite (002) reflection and $\beta_{\mathrm{intrumental}}^{2}$ is the FWHM obtained from the LaB_6_ standard. The corrected *β* value was then converted from degrees (2*θ*) to radians and used to estimate crystallite size according to the Scherrer equation:

$$\mathrm{Cs}=\frac{\mathrm{k\lambda}}{\beta \cos\;\theta }$$

where *Cs* is the hydroxyapatite crystallite size, *k* is the Scherrer constant (0.9), *λ* is the X-ray wavelength (1.5406 Å), *β* is the instrument-corrected FWHM expressed in radians, and *θ* is the Bragg angle corresponding to the (002) reflection.

### 2.11. Statistical Analysis

The results are presented as mean values ± standard deviation (SD). For end-point variables measured once per animal, the experimental treatment group was considered the single between-subject factor; therefore, comparisons among groups were performed using one-way analysis of variance (ANOVA), followed by Tukey’s post hoc test, with a significance level set at α = 0.05. Inferential statistical analyses were performed using GraphPad Prism version 7.02 (GraphPad Software Inc., San Diego, CA, USA).

For the principal biologically relevant pairwise comparisons, Tukey-adjusted *p*-values, 95% confidence intervals (95% CI) for the mean differences, and standardized effect sizes expressed as Hedges’ *g* were additionally reported. The principal comparisons were Ctrl versus Sham, Ctrl versus LMCC, LMCC versus LMMix, and LMCC versus LXCC. Hedges’ *g* was calculated separately from the group means, standard deviations, and sample sizes (*n* = 8 rats per group), whereas 95% confidence intervals were calculated for the corresponding mean differences. These additional effect-size and confidence-interval calculations were performed retrospectively from the available summary statistics and were not generated by GraphPad Prism version 7.02.

Initial and final body weights were analyzed separately using Welch’s ANOVA followed by Holm-adjusted pairwise comparisons. Food intake was analyzed using one-way ANOVA followed by Tukey’s multiple-comparison test, using individual animal records (*n* = 8 rats per group). Estimated calcium intake was calculated from the same food-intake measurements using the formulated dietary calcium concentration of 5 mg/g; therefore, the statistical comparisons were identical for both variables. Data distribution and the presence of potential outliers were evaluated by graphical inspection using box plots generated with Statgraphics Centurion version 19 (Statgraphics Technologies Inc., The Plains, VA, USA).

For serum estrogen and BMD, only group-level summary statistics (mean ± SD) were available for the baseline and final measurements, whereas the individual paired observations were not available for reanalysis. Consequently, a valid repeated-measures analysis, mixed-effects model, or baseline-adjusted ANCOVA could not be performed retrospectively. Baseline and final group summaries are therefore presented separately, and changes over time based on group means are interpreted descriptively rather than as paired within-animal comparisons.

Because no a priori power calculation was performed, a retrospective sensitivity analysis was conducted to evaluate the detectable effect size for the implemented design. Considering 10 experimental groups, a total sample size of 80 animals (*n* = 8 per group), a one-way ANOVA, α = 0.05, and statistical power of 0.80, the design was capable of detecting an omnibus effect size of approximately Cohen’s *f* = 0.47 (η^2^ ≈ 0.18). Thus, the study had adequate sensitivity to detect large treatment effects, whereas smaller effects may have remained undetected.

## 3. Results and Discussion

### 3.1. Characterization of the Nanocomplex Systems

The physicochemical characterization of the whey protein–polysaccharide nanocomplexes was performed to evaluate the structural features of the systems obtained by complex coacervation. Particle size, polydispersity index (PDI), ζ-potential, and electron microscopy were analyzed to compare the nanocomplexes formulated with *Opuntia ficus-indica* mucilage and *Tamarindus indica* xyloglucan and to determine the influence of polysaccharide type on their physicochemical organization.

#### 3.1.1. Particle Size, Polydispersity Index and ζ-Potential

The particle size distribution of the whey protein–polysaccharide nanocomplexes is shown in Figure 1. Whey protein–*Opuntia ficus-indica* mucilage nanocomplexes exhibited an average particle size of 213 ± 26 nm, whereas whey protein–*Tamarindus indica* xyloglucan nanocomplexes showed a larger average particle size of 314 ± 39 nm. These results indicate that both polysaccharides were able to form submicron protein–polysaccharide structures under the complex coacervation and ultrasonication conditions employed, although the type of polysaccharide influenced the dimensions of the resulting nanocomplexes.

The polydispersity index (PDI) was 0.29 ± 0.02 for the mucilage-based nanocomplexes and 0.32 ± 0.03 for the xyloglucan-based nanocomplexes. These values indicate moderately narrow particle-size distributions for both systems [33]. The slightly lower PDI observed for the mucilage-containing nanocomplexes suggests a somewhat more homogeneous particle population, which is consistent with the particle-size distribution profiles shown in Figure 1.

Differences between both nanocomplexes may be related to the distinct chemical characteristics of the polysaccharides. *O. ficus-indica* mucilage contains uronic acid residues, including galacturonic acid, whose carboxyl groups may become ionized depending on the pH of the system [34]. The carboxyl groups associated with these acidic residues may become ionized depending on the pH of the system and promote electrostatic interactions with positively charged regions of whey proteins, potentially favoring the formation of more compact protein–polysaccharide assemblies [27,35]. In addition, the highly hydrated and branched nature of the mucilage may contribute to steric stabilization of the resulting nanocomplexes [35]. In contrast, tamarind seed xyloglucan is predominantly a neutral polysaccharide composed mainly of a β-(1→4)-D-glucan backbone substituted with xylose and galactose residues and contains considerably fewer ionizable groups [10]. Consequently, its association with whey proteins may involve a greater contribution from hydrogen bonding, hydrophobic interactions, and polymer-chain entanglement [35]. This different interaction pattern may contribute to the formation of larger and slightly more heterogeneous nanocomplexes.

Regarding ζ-potential, the mucilage-based nanocomplexes exhibited a value of +18.2 ± 2.45 mV, whereas the xyloglucan-based nanocomplexes showed a value of +6.01 ± 1.78 mV. The greater absolute ζ-potential observed for the mucilage-containing system indicates a higher surface charge magnitude and suggests stronger electrostatic repulsion between particles than in the xyloglucan-based system [36]. Nevertheless, because the formulations were subsequently administered in dehydrated form, ζ-potential should be interpreted primarily as a physicochemical descriptor of the dispersed nanocomplexes rather than as a direct predictor of their stability under in vivo conditions.

Taken together, particle size, PDI, and ζ-potential indicate that the chemical nature of the polysaccharide influenced the physicochemical organization of the whey protein–polysaccharide nanocomplexes. The mucilage-based system formed smaller and slightly more homogeneous structures with a greater surface charge magnitude, whereas the xyloglucan-based system produced larger nanocomplexes with a lower surface charge magnitude. These physicochemical differences provide a basis for interpreting the distinct structural and biological responses observed for the two protein–polysaccharide formulations.

#### 3.1.2. Morphology of the Nanostructured System

Electron microscopy revealed distinct morphological features between the whey protein–*Opuntia ficus-indica* mucilage and whey protein–*Tamarindus indica* xyloglucan nanocomplexes (Figure 2).

The mucilage-based nanocomplexes exhibited predominantly rounded structures with relatively well-defined contours and a lower tendency to aggregate (Figure 2a). In contrast, the xyloglucan-based nanocomplexes showed a more aggregated morphology, with partially coalesced structures and less clearly defined boundaries (Figure 2b). These morphological observations were consistent with the particle-size and polydispersity results described above. The mucilage-based system, which exhibited a smaller average particle size and slightly lower PDI, also showed a more uniform structural organization, whereas the larger particle size and slightly greater heterogeneity of the xyloglucan-based system were reflected in its more aggregated appearance. This agreement between electron microscopy and dynamic light scattering supports the interpretation that the type of polysaccharide influenced the architecture of the resulting protein–polysaccharide nanocomplexes [35].

Taken together with the ζ-potential results, the morphological observations indicate that the mucilage-based formulation exhibited a more homogeneous structural organization than the xyloglucan-based formulation. Although the present microscopy analysis does not establish the molecular mechanisms responsible for these differences, the combined physicochemical characterization consistently demonstrates that polysaccharide composition influenced the structural organization of the whey protein–polysaccharide nanocomplexes.

### 3.2. Validation of the Ovariectomized Rat Model

Serum estrogen levels were evaluated to verify the establishment of estrogen deficiency following ovariectomy. At the beginning of the experimental period, the Sham group showed a higher mean serum estrogen concentration (53.33 ± 0.96 pg/mL) than the ovariectomized animals (13.33 ± 0.17 pg/mL). A similar between-group difference was observed at the end of the experimental period, with values of 50.66 ± 0.73 pg/mL in the Sham group and 9.27 ± 0.23 pg/mL in the ovariectomized animals (*p* ≤ 0.05 for between-group comparisons at each time point). These group-level findings are consistent with the expected reduction in circulating estrogen following bilateral ovariectomy and support the establishment of an estrogen-deficient OVX model [12,37]. Because individual paired estrogen measurements were not available for reanalysis, changes between baseline and final values are interpreted descriptively rather than as within-animal longitudinal effects.

### 3.3. Body Weight, Food Intake, and Estimated Calcium Intake

Initial and final body weight, estimated daily food intake, and estimated calcium intake are presented in Table 2. Body weight varied among the experimental groups at both the beginning and the end of the study. Importantly, differences in body weight were already apparent at baseline; therefore, differences in final body weight cannot be attributed exclusively to the dietary interventions. Estimated food intake was numerically lower in the CCa and MOF groups, whereas Ctrl, Whey, XLG, LXCC, LXMix, LMCC, and LMMix showed similar estimated daily intakes, ranging from 30.23 to 30.43 g/rat/day. The corresponding estimated calcium intakes in these latter groups ranged from 151.17 to 152.15 mg/rat/day. These measurements provide nutritional context for interpreting the bone-quality outcomes described in the following sections.

These findings are relevant to the interpretation of bone outcomes because differences in food consumption may have resulted in differences in estimated calcium intake among groups, despite the use of the same formulated dietary calcium concentration. In particular, the lower estimated calcium intake in the CCa and MOF groups should be considered when interpreting their bone responses. Conversely, the similar estimated calcium intakes in the Ctrl, Whey, XLG, LXCC, LXMix, LMCC, and LMMix groups provide nutritional context for comparisons of their bone outcomes. Nevertheless, because calcium intake was estimated from the dietary formulation rather than determined by chemical analysis of the consumed diets, these results cannot establish equivalent actual calcium exposure or differences in calcium absorption.

### 3.4. Femoral Morphometric and Biomechanical Properties

The physical dimensions of rat femurs are summarized in Table 3. Compared with the sham group, ovariectomy did not significantly affect femoral length, weight, width, or thickness. These findings are consistent with previous reports indicating that estrogen deficiency has a greater impact on bone quality and remodeling than on gross skeletal dimensions. Regarding this, Hsu et al. [38] reported no significant alterations in femoral and mandibular measurements following ovariectomy, while Kaczmarczyk-Sedlak et al. [39] similarly observed no marked changes in bone length, mass, or width in ovariectomized rats. A greater increase in tibial length has been reported in ovariectomized rodents compared with sham-operated animals (3.7% vs. 2.5%, respectively), although these differences were not statistically significant, suggesting that estrogen deficiency exerts only limited effects on longitudinal bone growth in rodent models [40]. This phenomenon has been attributed to the continued activity of growth plates in rodents, in contrast to humans, where epiphyseal closure occurs before menopause [41,42].

Although no statistically significant differences were detected among treatments, some groups exhibited numerically higher values for specific dimensional parameters. The LXCC group showed the greatest femoral length, MOF exhibited the highest bone weight, LMMix presented the largest femoral width, and CCa showed the highest thickness values. Overall, these results suggest that the experimental treatments did not substantially modify gross femoral morphology under the conditions evaluated.

Bone mechanical strength was assessed by compression (Fmax) and three-point bending (Pmax) tests, which are widely recognized as reliable indicators of bone quality and fracture resistance [43]. The ovariectomized control group exhibited the lowest values for both Fmax and Pmax, indicating impaired mechanical competence under estrogen-deficient conditions. Significant differences (*p* ≤ 0.05) were observed between the control group and all experimental treatments. These findings are consistent with previous reports demonstrating that estrogen deficiency compromises bone mechanical performance and increases skeletal fragility. Chen et al. [44], in a systematic review of ovariectomized rat models, concluded that ovariectomy-induced bone loss reduced biomechanical properties and delayed fracture healing, highlighting the sensitivity of biomechanical testing for evaluating skeletal deterioration.

Groups CCa, LMMix, LXCC, LXMix, and XLG required between 520 and 530 N to induce fracture during compression testing, whereas MOF, Sham, and Whey exhibited values ranging from 537 to 566 N. Notably, LMCC exhibited the highest Fmax value (583.10 ± 10.8 N). Compared with Ctrl, the mean difference was 99.40 N (95% CI: 80.83–117.97; Tukey-adjusted *p* < 0.0001; Hedges’ g = 6.74). LMCC also showed higher Fmax than LMMix (mean difference: 54.00 N, 95% CI: 35.43–72.57; *p* < 0.0001; Hedges’ g = 5.07) and LXCC (mean difference: 62.30 N, 95% CI: 43.73–80.87; *p* < 0.0001; Hedges’ g = 5.61).

A similar trend was observed in the three-point bending test, where the control group exhibited the lowest resistance and LMCC showed the highest Pmax value. For Pmax, LMCC exceeded Ctrl by 58.90 N (95% CI: 48.63–69.17; Tukey-adjusted *p* < 0.0001; Hedges’ g = 5.43), LMMix by 50.20 N (95% CI: 39.93–60.47; *p* < 0.0001; Hedges’ g = 5.17), and LXCC by 22.40 N (95% CI: 12.13–32.67; *p* = 3.26 × 10^−8^; Hedges’ g = 2.28). Compared with the ovariectomized control group, LMCC increased Fmax by approximately 20% and Pmax by approximately 80%, indicating a substantial improvement in bone mechanical performance. The more favorable biomechanical response observed in LMCC compared with the corresponding physical mixture suggests that formulation strategy and structural organization may be associated with differences in biomechanical performance [45].

An interesting finding was the different biomechanical performance of the nanostructured formulations compared with their corresponding physical mixtures. LMCC exhibited greater mechanical resistance than LMMix, while LXCC showed superior biomechanical performance compared with LXMix; however, this advantage was not consistently observed across all evaluated bone-quality parameters. These findings indicate that formulation strategy and structural organization may be associated with differences in biomechanical response. Protein–polysaccharide complexation has been reported to modify the physicochemical properties of biopolymers [45]; however, the specific mechanisms responsible for the differences observed in the present study were not directly investigated and therefore remain to be established.

According to Table 2, estimated calcium intake was similar in LMCC and LMMix (151.46 and 152.15 mg/rat/day, respectively), as well as in LXCC and LXMix (151.17 and 151.95 mg/rat/day, respectively). Therefore, the observed differences in biomechanical performance between the corresponding nanocomplex and physical-mixture groups are unlikely to be explained simply by differences in estimated dietary calcium intake. However, these estimates do not establish equivalent analytically measured calcium exposure or demonstrate enhanced calcium absorption.

These results suggest that the structural organization of protein–polysaccharide complexes may play an important role in determining their biological effectiveness. Protein–polysaccharide complexation has been reported to modify the functional properties of biopolymers, improving their stability, solubility, and structural organization compared with the individual components. Such characteristics may contribute to the superior biomechanical performance observed in the nanostructured formulations evaluated in the present study [45]. However, the mechanisms underlying these effects require further investigation.

Overall, the biomechanical values obtained in this study fall within the range previously reported for rats of similar age and strain, supporting the reliability of the experimental model and testing methodologies employed [38,39,46]. Moreover, the greater sensitivity of biomechanical parameters compared with gross femoral dimensions suggests that bone mechanical properties may represent an earlier and more responsive indicator of treatment-related changes in skeletal integrity under estrogen-deficient conditions.

### 3.5. Femoral Calcium and Phosphorus Contents and Ca/P Ratio

Compared with the sham group, ovariectomy significantly reduced femoral calcium content, confirming the detrimental effect of estrogen deficiency on bone mineralization (Figure 3). The OVX control group exhibited approximately 30% lower calcium levels than the sham group (*p* ≤ 0.05). All dietary treatments attenuated calcium loss to some extent; however, the LMCC group showed the highest femoral calcium content and significantly higher calcium concentrations than the OVX control group and most experimental treatments (*p* ≤ 0.05). Compared with Ctrl, LMCC showed 7.54 percentage points higher femoral calcium content (95% CI: 7.09–7.99; Tukey-adjusted *p* < 0.0001; Hedges’ g = 25.01). LMCC also showed higher femoral calcium content than LMMix (mean difference: 2.17 percentage points, 95% CI: 1.72–2.62; *p* < 0.0001; Hedges’ g = 6.93) and LXCC (mean difference: 3.54 percentage points, 95% CI: 3.09–3.99; *p* < 0.0001; Hedges’ g = 11.00). These findings indicate that the LMCC group exhibited higher femoral calcium content than the untreated ovariectomized control and most experimental treatment groups under estrogen-deficient conditions. These results are similar to those reported by Quintero-García et al. [12], who observed that ovariectomized rats showed reduced femoral calcium content, whereas supplementation with *Opuntia ficus-indica* mucilage was associated with higher femoral calcium content and more favorable bone-related parameters. Interestingly, the LMCC group exhibited higher femoral calcium content than the corresponding LMMix group, whereas the LXMix group exhibited higher femoral calcium content than LXCC. Thus, the effect of nanostructuring on femoral calcium content was not uniform across the two polysaccharide systems. These contrasting responses suggest that formulation composition and physicochemical organization may be associated with differences in femoral calcium content; however, calcium association within the nanostructures, calcium release, gastrointestinal bioaccessibility, and intestinal absorption were not measured in the present study. Consequently, the mechanisms underlying these differences cannot be established from the current data.

According to Table 2, estimated calcium intake was similar in LMCC and LMMix (151.46 and 152.15 mg/rat/day, respectively). Thus, the higher femoral calcium content observed in LMCC cannot be readily attributed to a greater estimated dietary calcium intake. Nevertheless, because calcium intake was estimated from the dietary formulation, this comparison does not establish equivalent actual calcium exposure or demonstrate enhanced intestinal calcium absorption.

In contrast, the LXMix group exhibited higher femoral calcium content than the LXCC group, further indicating that nanostructuring did not uniformly increase femoral calcium content across the two polysaccharide systems.

Also, phosphorus concentrations exhibited significant differences (*p* ≤ 0.05) among experimental groups, indicating that phosphorus homeostasis was also affected by ovariectomy and dietary intervention. The marked phosphorus depletion detected in the OVX control group contributed substantially to alterations in the mineral profile of femoral bone. It is widely reported that estrogen deficiency increases osteoclast activity (bone resorption) in relation to osteoblast activity (bone formation) in human and rodents. This promotes the release of Ca and P from the bone into the blood stream, where it is then excreted in urine [47]. Nevertheless, the similar patterns observed for Ca and P content in femoral bone confirmed the co-dependence and interrelationship of these minerals in bone development as components of hydroxyapatite (HA), the major mineral constituent of bone, as previously reported [48].

Bone mineral density is widely accepted as an indicator of skeletal health; nevertheless, several authors have highlighted the importance of complementary mineral composition analyses to achieve a more comprehensive evaluation of bone quality. In this regard, the Ca/P ratio has been proposed as an additional indicator of bone mineral status and mineral phase composition [39,46]. Previous studies have reported Ca/P values ranging from approximately 1 to 3 in different rat strains and ages [49]. In the present study, Ca/P ratios in all groups except the OVX control remained within this interval; however, significant differences among treatments were observed.

Interestingly, the OVX control group exhibited the highest Ca/P ratio, whereas the sham group and several treated groups showed substantially lower values. A similar trend was reported by Quintero-García et al. [12], who observed elevated Ca/P values in ovariectomized rats despite reduced calcium concentrations. These findings suggest that changes in the Ca/P ratio may not exclusively reflect calcium depletion but rather differential modifications in both calcium and phosphorus content. Because the Ca/P ratio is determined by the relative abundance of both minerals, disproportionate losses of either component may significantly alter this parameter. HA is composed of calcium (Ca) and phosphorus (P); consequently, the bone Ca/P molar ratio can be used as an indicator of bone mineral composition and quality. Biological hydroxyapatite is calcium-deficient compared with stoichiometric hydroxyapatite; therefore, its theoretical Ca/P molar ratio is approximately 1.67 [50,51]. Based on previous evidence, Kato et al. [52] highlighted that alterations in the elemental composition of bone may occur despite an apparently normal bone mineralization density distribution, suggesting that changes in calcium and phosphorus content may contribute to skeletal fragility independently of mineralization density. This concept may explain the elevated Ca/P ratio observed in the OVX group, whereas the remaining experimental groups exhibited values below 2.0.

The Ca/P ratios observed in the present study followed a trend comparable to that reported by Quintero-García et al. [12], particularly regarding the elevated values observed in ovariectomized animals and the lower ratios detected in mucilage-treated groups, supporting the interpretation that the mucilage-treated groups exhibited a more balanced bone mineral composition under the conditions evaluated. Interestingly, previous studies have reported conflicting effects of ovariectomy on the Ca/P ratio. While Quintero-García et al. [12] observed increased Ca/P values in ovariectomized rats, Helder-Fonseca et al. [46] and Kaczmarczyk-Sedlak et al. [39] reported lower ratios following ovariectomy. Such discrepancies may reflect differences among experimental models and analytical approaches used to evaluate bone mineral composition [40,44,52].

The biological significance of these differences remains unclear; however, alterations in Ca/P stoichiometry may influence mineral organization and, consequently, bone mechanical competence. Notably, the LMCC group combined relatively high femoral calcium content with a Ca/P ratio closer to that observed in healthy animals, suggesting a more favorable mineral composition. Although stoichiometric hydroxyapatite exhibits a Ca/P molar ratio of 1.67, biological bone mineral is a non-stoichiometric apatite whose composition is influenced by carbonate substitution, ionic deficiencies, and structural heterogeneity. Consequently, variations in Ca/P ratio have been reported among different biological tissues and physiological conditions [53,54].

Finally, the higher calcium content observed in the LMCC group was consistent with the superior biomechanical performance previously described for this treatment. Although causality cannot be established from the present results alone, the higher femoral calcium content observed in the LMCC group was consistent with its more favorable biomechanical performance under estrogen-deficient conditions.

### 3.6. Femoral Bone Microarchitecture

Bone quality encompasses bone material properties, including bone microarchitecture, mineralization, material composition, bone turnover, microdamage accumulation, and other structural characteristics that collectively determine skeletal strength beyond bone mineral density (BMD) alone [55]. Within this context, scanning electron microscopy (SEM), together with quantitative histomorphometric analysis (Ct.Th, Tb.Th, and Tb.Sp), enabled a comprehensive evaluation of femoral bone microarchitecture. Representative SEM micrographs demonstrated that ovariectomy induced marked deterioration of trabecular and cortical bone architecture (Figure 4a–c), whereas the experimental treatments preserved these structural features to different extents (Figure 4d–r). These qualitative observations were consistent with the quantitative histomorphometric findings (Figure 5a–c), providing complementary evidence of the structural modifications induced by each treatment. Therefore, SEM and histomorphometric analyses complement conventional densitometric evaluation by providing structural information that cannot be captured by BMD measurements alone [56]. Furthermore, cortical bone thickness has been positively associated with mechanical competence, as reduced cortical thickness increases stress and strain concentrations within the femur, thereby predisposing bone to structural failure [57].

Representative SEM micrographs revealed that ovariectomy significantly (*p* < 0.05) altered the femoral bone microarchitecture, as evidenced by trabecular thinning, increased trabecular separation, disruption of trabecular connectivity, and cortical thinning in the Ctrl group (Figure 4a–c) compared with the Sham group (Figure 4j–l). These qualitative observations were corroborated by quantitative histomorphometric analysis, which demonstrated reduced trabecular thickness (Tb.Th) and cortical thickness (Ct.Th), together with increased trabecular separation (Tb.Sp), confirming the deleterious effects of estrogen deficiency on bone microarchitecture (Figure 5a–c). This structural deterioration is consistent with the molecular mechanisms underlying postmenopausal osteoporosis, in which estrogen deficiency promotes osteoclastogenesis through increased RANKL signaling, suppresses osteoblastic bone formation, and enhances bone remodeling, resulting in accelerated trabecular bone loss because of its higher metabolic activity and remodeling rate [58]. Consequently, deterioration of the trabecular network is considered one of the earliest manifestations of estrogen-deficiency-induced bone loss and has been shown to predict the subsequent progression of skeletal deterioration [59].

Among the experimental treatments evaluated, LMCC exhibited the greatest preservation of both trabecular and cortical bone microarchitecture, although its structural characteristics remained inferior to those observed in the Sham group. Representative SEM micrographs showed thicker and more continuous trabeculae, reduced trabecular separation, and better preservation of cortical bone in the LMCC group than in the other ovariectomized groups (Figure 4d–f). These qualitative observations were consistent with the quantitative histomorphometric analysis (Figure 5), LMCC exhibited the most favorable microarchitectural profile among the ovariectomized groups (*p* < 0.05). For cortical thickness (Ct.Th), LMCC showed higher values than Ctrl (mean difference: 94.48 µm, 95% CI: 77.56–111.40; Tukey-adjusted *p* < 0.0001; Hedges’ g = 11.30), LMMix (mean difference: 45.69 µm, 95% CI: 28.77–62.61; *p* < 0.0001; Hedges’ g = 3.89), and LXCC (mean difference: 87.15 µm, 95% CI: 70.23–104.07; *p* < 0.0001; Hedges’ g = 9.29). For trabecular thickness (Tb.Th), LMCC also showed higher values than Ctrl (mean difference: 36.33 µm, 95% CI: 22.34–50.32; *p* < 0.0001; Hedges’ g = 4.49), LMMix (mean difference: 17.24 µm, 95% CI: 3.25–31.23; *p* = 0.0052; Hedges’ g = 1.71), and LXCC (mean difference: 21.25 µm, 95% CI: 7.26–35.24; p = 0.00019; Hedges’ g = 2.52). In contrast, for trabecular separation (Tb.Sp), LMCC showed lower values than Ctrl (mean difference: −4.65 µm, 95% CI: −6.60 to −2.70; *p* < 0.0001; Hedges’ g = −5.70) and LXCC (mean difference: −2.55 µm, 95% CI: −4.50 to −0.60; *p* = 0.0022; Hedges’ g = −2.43), whereas the difference between LMCC and LMMix was not statistically significant (mean difference: −1.81 µm, 95% CI: −3.76 to 0.14; *p* = 0.0898; Hedges’ g = −1.67).

Although LMCC showed the most favorable microarchitectural profile among the ovariectomized groups, the mechanisms responsible for this response were not directly investigated. The physicochemical characterization demonstrated differences between the mucilage- and xyloglucan-based nanostructures in particle size, polydispersity, surface charge, and morphology, suggesting that formulation composition and structural organization may be relevant to the observed biological responses. However, the present study did not evaluate calcium release, gastrointestinal bioaccessibility, intestinal absorption, gut microbiota, or bone-remodeling biomarkers. Therefore, any relationship between these mechanisms and the observed preservation of trabecular and cortical architecture should be regarded as hypothetical and requires direct experimental verification.

Collectively, these findings suggest that preservation of trabecular and cortical architecture was consistent with the more favorable biomechanical properties observed in the Sham and LMCC groups (Table 3).

While all the experimental treatments were associated with different microarchitectural response patterns. Treatments containing individual functional ingredients showed less favorable preservation of trabecular and cortical architecture, whereas the corresponding physical mixtures showed intermediate responses. (Figure 5a–c). In contrast, the LMCC nanostructured formulation exhibited the most favorable microarchitectural profile among the ovariectomized groups. These findings indicate that formulation strategy and physicochemical organization may be associated with the observed differences; however, the underlying mechanism was not directly investigated.

Although the mechanisms underlying these observations remain to be fully elucidated, future studies should investigate the biological processes responsible for the superior structural preservation observed in the LMCC group. Although intestinal calcium absorption was not directly evaluated, the higher femoral calcium content and the more favorable Ca/P ratio observed in the LMCC group were consistent with its more favorable microarchitectural profile (Figure 3a,b). Taken together, these findings show concordance among femoral mineral content, bone microarchitecture, and biomechanical performance in the LMCC group, although causal relationships among these outcomes cannot be established from the present study.

The more favorable trabecular and cortical architecture observed in the LMCC group provides a structural context for interpreting the corresponding BMD and biomechanical findings. Because bone microarchitecture, mineralization, and mechanical properties represent complementary aspects of bone quality, the concordant pattern observed across these outcomes suggests an association among structural preservation, mineral status, and mechanical competence under estrogen-deficient conditions. However, the present study does not establish causal relationships among these variables.

### 3.7. Bone Mineral Density

Femoral bone mineral density (BMD) was evaluated at the beginning and at the end of the experimental period (Figure 6). At baseline, the experimental groups showed comparable group mean BMD values. At the end of the study, the Sham group exhibited the highest mean BMD, whereas the untreated ovariectomized control group showed the lowest final value. The supplemented groups showed intermediate final BMD values, with LMCC exhibiting the highest final BMD among the supplemented ovariectomized groups and approaching the value observed in the Sham group. Between-group differences at the final measurement were statistically significant (*p* < 0.05). At the final assessment, LMCC showed a higher BMD than Ctrl (mean difference: 0.25 g/cm^2^, 95% CI: 0.211–0.289; Tukey-adjusted *p* < 0.0001; Hedges’ g = 9.27). LMCC also showed higher final BMD than LMMix (mean difference: 0.040 g/cm^2^, 95% CI: 0.001–0.079; *p* = 0.0433; Hedges’ g = 1.89) and LXCC (mean difference: 0.070 g/cm^2^, 95% CI: 0.031–0.109; *p* < 0.0001; Hedges’ g = 4.19).

Descriptively, comparison of the group means between baseline and the end of the experimental period indicated an increase of approximately 22% in the Sham group and increases of approximately 10–13% in the treated ovariectomized groups. Because individual paired BMD observations were not available for reanalysis, these changes over time are presented descriptively and should not be interpreted as statistically tested within-animal longitudinal effects. The final BMD pattern was consistent with the differences observed in bone microarchitecture, femoral mineral content, and biomechanical properties.

The more favorable response observed for LMCC may be associated with the specific composition and physicochemical organization of the whey protein–mucilage formulation. *O. ficus-indica* mucilage contains uronic acid residues, whereas tamarind xyloglucan is predominantly composed of neutral sugars; these compositional differences may influence protein–polysaccharide interactions and the resulting nanostructure [10,34,60]. However, calcium association within the nanostructures, calcium-release kinetics, gastrointestinal bioaccessibility, and intestinal calcium absorption were not evaluated in the present study. Therefore, these mechanisms cannot be invoked as direct explanations for the observed BMD differences and should be investigated in future studies.

The densitometric findings were highly consistent with the remaining biological and physicochemical parameters evaluated in the present study. Consistently, the untreated ovariectomized control exhibited not only the lowest final BMD but also the lowest femoral calcium content, the highest Ca/P ratio, and the poorest biomechanical performance, whereas Sham animals consistently displayed the opposite pattern. Among the ovariectomized groups, LMCC showed an overall response pattern closer to that of the Sham group across several evaluated variables, showing that the more favorable final BMD values in LMCC were accompanied by a more favorable pattern in femoral mineral content and biomechanical performance. Interestingly, the effect of formulation strategy differed between the two polysaccharide systems. In the mucilage-based treatments, LMCC generally showed a more favorable response than the corresponding physical mixture, whereas within the xyloglucan-based treatments, LXMix exhibited greater BMD than LXCC, although LXCC showed advantages in selected parameters such as biomechanical performance. These findings indicate that nanostructuring did not confer a uniform biological advantage independently of polysaccharide type.

According to Table 2, estimated calcium intake was comparable between LMCC and LMMix (151.46 and 152.15 mg/rat/day, respectively) and between LXCC and LXMix (151.17 and 151.95 mg/rat/day, respectively). Thus, the different BMD responses observed within these formulation pairs are unlikely to be explained simply by differences in estimated dietary calcium intake. These comparisons support consideration of formulation-dependent effects, although they do not establish equivalent analytically measured calcium exposure or demonstrate enhanced calcium absorption.

Rather, the biological response appears to depend on the specific physicochemical organization generated by each protein–polysaccharide system, supporting a formulation-dependent structure–function relationship.

Overall, LMCC showed a more favorable pattern than LXCC across most of the evaluated bone-related outcomes. However, the present study does not establish the causal mechanism responsible for this differential response, and the possible contribution of formulation composition and physicochemical organization should therefore be considered a hypothesis requiring direct experimental verification.

### 3.8. Bone Mineral Crystallinity

X-ray diffraction (XRD) analysis was performed to evaluate differences in the crystallographic characteristics of bone-derived hydroxyapatite among the experimental groups [61]. The diffraction patterns obtained from femoral bone samples (Figure 7a) exhibited the characteristic reflections of biological hydroxyapatite in all experimental groups, confirming that hydroxyapatite remained the predominant mineral phase irrespective of ovariectomy or nutritional treatment. No additional diffraction peaks corresponding to secondary crystalline phases were detected, indicating that the interventions did not modify the crystalline identity of bone mineral. Although the diffraction profiles were qualitatively similar among groups, analysis of the (002) reflection revealed differences in the crystallographic characteristics of bone-derived hydroxyapatite (Figure 7b).

Analysis of the full width at half maximum (FWHM) of the (002) hydroxyapatite reflection revealed significant differences among treatments (Figure 7b). The untreated ovariectomized Ctrl group exhibited the narrowest diffraction peak and the largest calculated crystallite size, whereas the Sham group showed the broadest diffraction peak and the smallest crystallite size. Among the ovariectomized treatment groups, LMCC exhibited a broader diffraction peak and a smaller crystallite size than LXCC, whereas the remaining treatment groups displayed intermediate values. Collectively, these findings indicate that the protein–polysaccharide formulations were associated with differences in the crystallographic characteristics of bone-derived hydroxyapatite and with distinct patterns of bone-mineral structural organization among the experimental groups. For FWHM, LMCC showed a higher value than Ctrl (mean difference: 0.1105° 2θ, 95% CI: 0.0938–0.1273; Tukey-adjusted *p* < 0.0001; Hedges’ g = 7.28), LMMix (mean difference: 0.0480° 2θ, 95% CI: 0.0313–0.0648; *p* < 0.0001; Hedges’ g = 3.10), and LXCC (mean difference: 0.0822° 2θ, 95% CI: 0.0655–0.0989; *p* < 0.0001; Hedges’ g = 5.53).

The biological significance of hydroxyapatite crystallite size should be interpreted within the physiological and pathological context of bone remodeling rather than as an isolated crystallographic parameter. As previously reported, bone mechanical competence does not depend simply on maximizing or minimizing crystallite size but rather on maintaining an appropriate distribution of mineral crystal dimensions throughout the continuous processes of bone formation, maturation, and remodeling [62]. Nevertheless, in osteoporotic bone, larger hydroxyapatite crystallites have frequently been associated with increased bone fragility and altered mineral organization, whereas non-osteoporotic bone typically exhibits a broader distribution of crystallite sizes that reflects the coexistence of newly formed and mature mineral crystals. Consistent with this concept, Noor et al. [63] reported that osteoporotic bone exhibited larger hydroxyapatite crystallites than normal bone, together with increased porosity and microstructural deterioration. In agreement with these reports, the untreated ovariectomized Ctrl group in the present study exhibited the largest crystallite size, which coincided with the lowest femoral bone mineral density, reduced bone calcium content, an altered Ca/P ratio, deteriorated trabecular microarchitecture, and inferior biomechanical performance. Conversely, the Sham group and, particularly, the LMCC group exhibited smaller crystallite sizes together with preservation of these complementary indicators of bone quality, suggesting that the crystallographic changes observed in the present study reflected differences in bone mineral organization associated with the underlying bone remodeling status rather than crystallite size alone.

A possible physicochemical context for these crystallographic differences is the mineral composition of femoral bone, particularly the Ca/P ratio. Hydroxyapatite crystal growth can be affected by the incorporation and substitution of mineral ions within the crystal lattice, and previous studies have shown that mono- and divalent ions may modify hydroxyapatite crystallite size, shape, and orientation [64]. In the present study, differences in phosphorus content and Ca/P ratio were observed among the experimental groups and were accompanied by differences in hydroxyapatite crystallite size. The untreated ovariectomized Ctrl group showed a more altered Ca/P balance together with the largest crystallite size, whereas LMCC exhibited a more balanced mineral composition together with a smaller crystallite size and a crystallographic pattern closer to that observed in Sham animals. However, ion substitution within the hydroxyapatite lattice was not directly evaluated, and the present results do not establish a causal mechanism linking mineral composition with crystallite size. Therefore, the observed associations should be interpreted as descriptive and require direct experimental verification.

## 4. Conclusions

In this ovariectomized rat model, whey protein–polysaccharide formulations were associated with differential effects on bone-related outcomes under estrogen-deficient conditions. Among the evaluated treatments, the whey protein–*Opuntia ficus-indica* mucilage nanostructured formulation (LMCC) showed a more favorable overall pattern across most of the evaluated bone-related outcomes, including bone mineral density, femoral calcium content, bone microarchitecture, hydroxyapatite crystallite size, and biomechanical properties. However, treatment effects were parameter-dependent, and the response to nanostructuring was not uniform across the two polysaccharide systems.

The mucilage-based nanostructured formulation generally showed a more favorable response than the xyloglucan-based nanostructured formulation, whereas the xyloglucan system exhibited a more variable pattern depending on the specific bone parameter assessed. These findings indicate that the biological response was associated with formulation composition and physicochemical organization; however, the mechanisms underlying these differences were not directly established in the present study.

Overall, the present findings support the preclinical relevance of whey protein–*O. ficus-indica* mucilage nanostructured formulations in relation to bone-related outcomes under estrogen-deficient conditions in ovariectomized rats. These results should not be extrapolated directly to osteoporosis prevention in humans. Further studies are required to determine calcium association within the nanostructures, calcium-release kinetics, gastrointestinal bioaccessibility, intestinal calcium absorption, bone-remodeling biomarkers, and the translational relevance and safety of these formulations before considering potential applications in human nutrition.

## Figures and Tables

**Figure 1:**
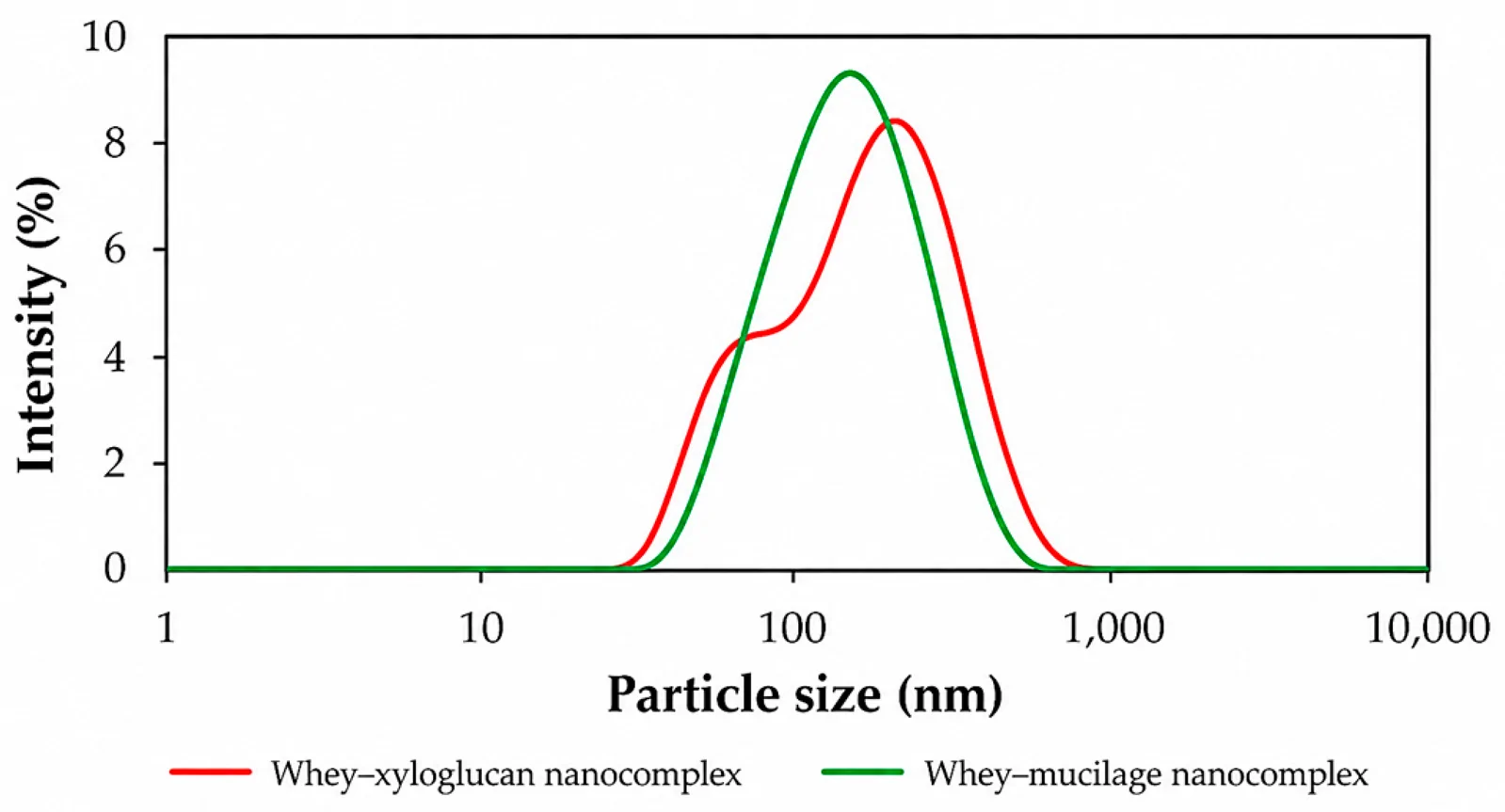
Particle-size distribution of whey protein–*Opuntia ficus-indica* mucilage and whey protein–*Tamarindus indica* xyloglucan nanocomplexes.

**Figure 2:**
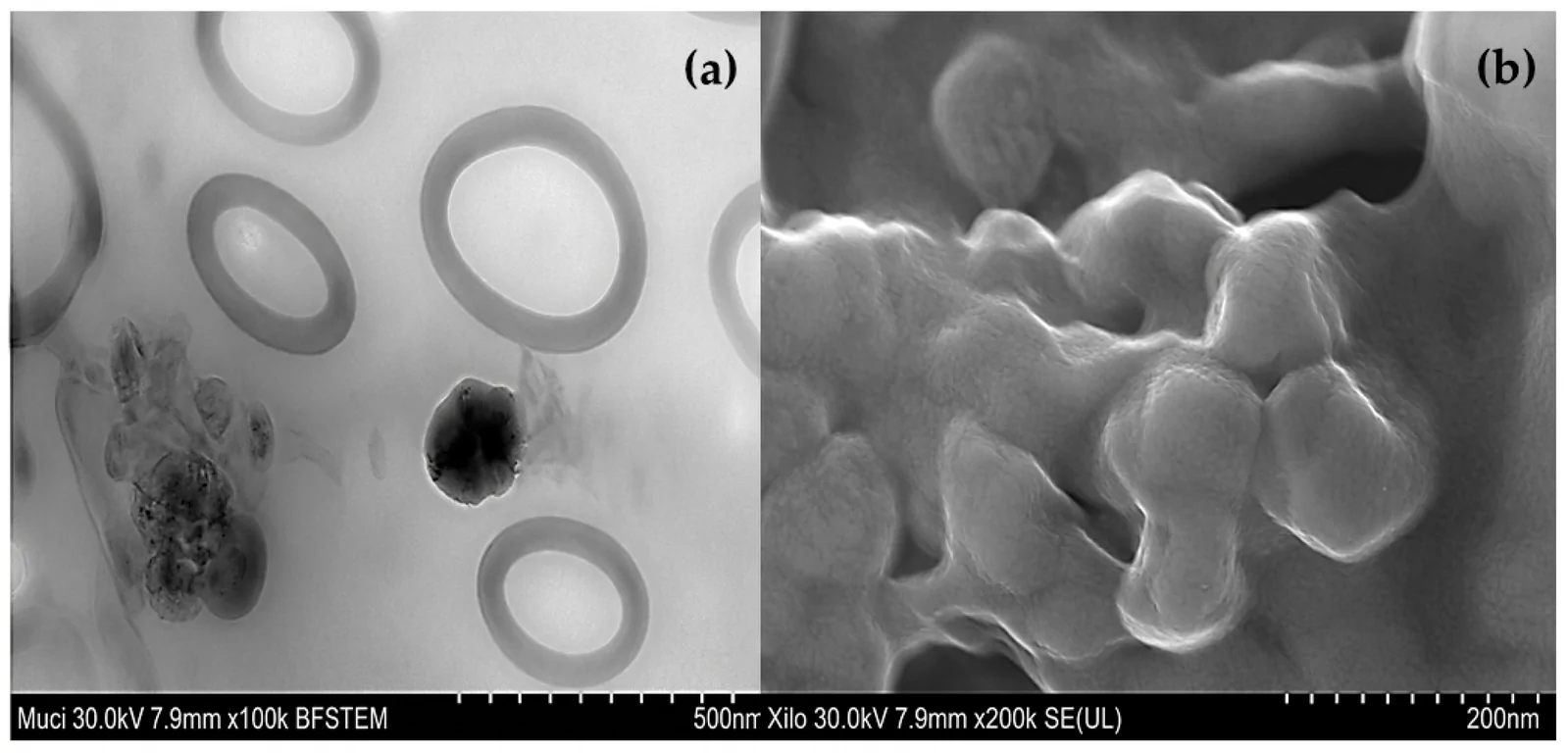
Representative electron micrographs of whey protein–mucilage (**a**) and whey protein–xyloglucan (**b**) nanocomplexes. The mucilage-based nanocomplex was imaged in bright-field scanning transmission electron microscopy (BF-STEM) mode at ×100,000 magnification (scale bar = 500 nm), whereas the xyloglucan-based nanocomplex was imaged using secondary-electron imaging [SE(UL)] at ×200,000 magnification (scale bar = 200 nm).

**Figure 3:**
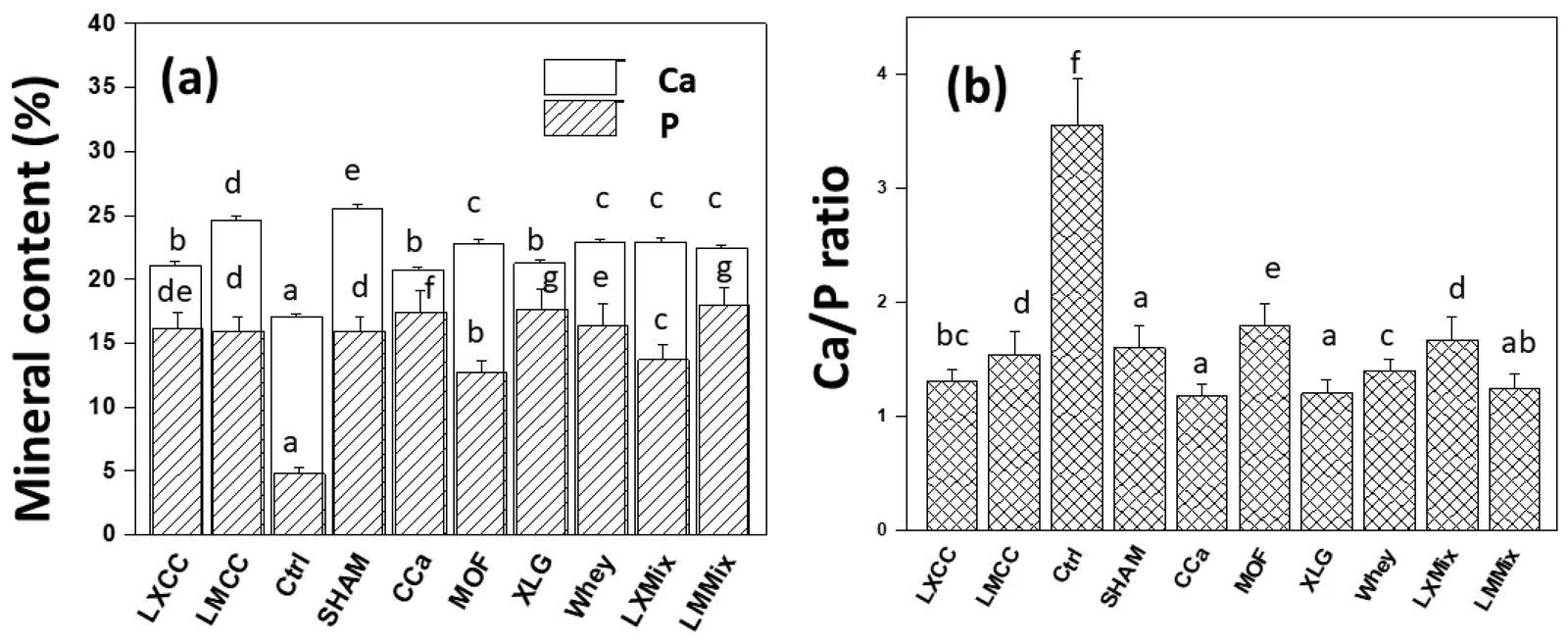
Calcium and phosphorus content and Ca/P ratio in femoral bone of ovariectomized rats subjected to the experimental treatments. (**a**) Femoral calcium and phosphorus content. (**b**) Femoral Ca/P ratio. Data are presented as mean ± SD (*n* = 8 per group). Different lowercase letters indicate statistically significant differences among experimental groups (*p* < 0.05), as determined by one-way ANOVA followed by Tukey’s multiple comparison test.

**Figure 4:**
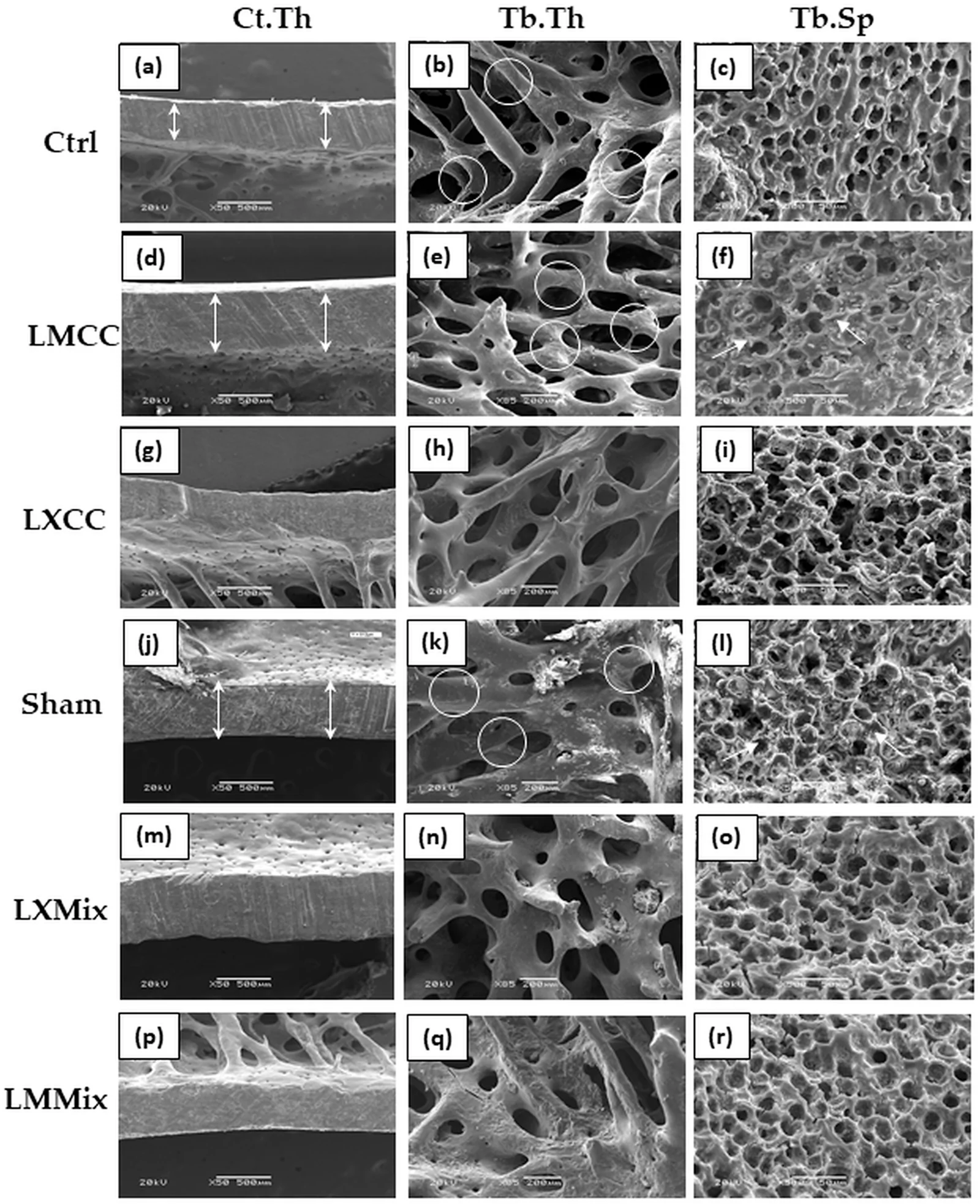
Representative SEM micrographs illustrating the histomorphometric parameters evaluated in femoral bone from the experimental groups. Representative images correspond to Ctrl (**a**–**c**), LMCC (**d**–**f**), LXCC (**g**–**i**), Sham (**j**–**l**), LXMix (**m**–**o**), and LMMix (**p**–**r**). Within each group, panels show cortical thickness (Ct.Th), trabecular thickness (Tb.Th), and trabecular separation (Tb.Sp), respectively. White double-headed arrows indicate Ct.Th, white circles indicate Tb.Th, and white arrows indicate Tb.Sp. The selected groups represent the range of microarchitectural changes observed across the experimental treatments.

**Figure 5:**
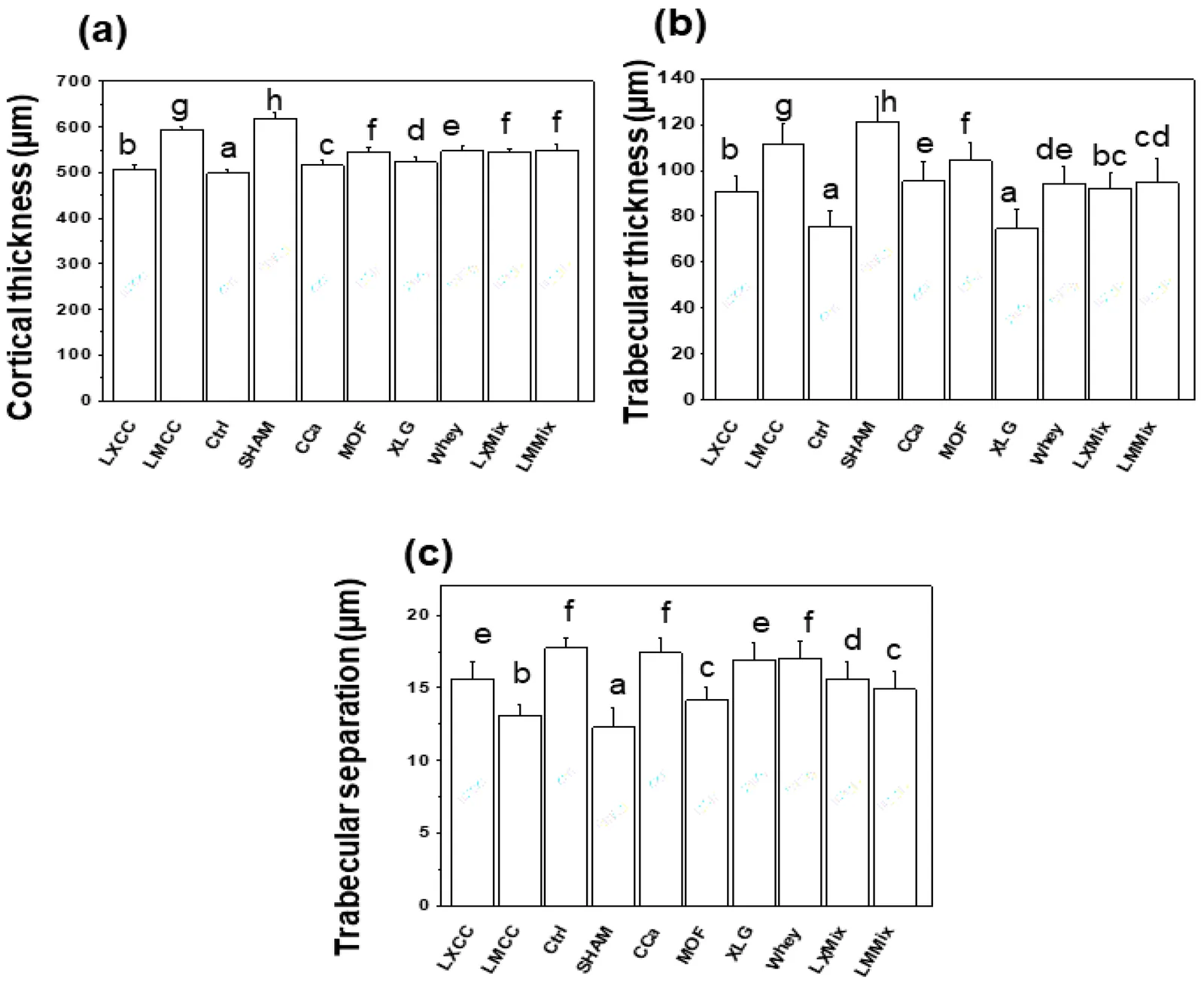
Histomorphometric evaluation of femoral bone microarchitecture in ovariectomized rats receiving the experimental treatments. (**a**) Cortical thickness (Ct.Th), (**b**) trabecular thickness (Tb.Th), and (**c**) trabecular separation (Tb.Sp) determined by histomorphometric analysis. Data are presented as mean ± SD (*n* = 8 per group). Different lowercase letters above the bars indicate statistically significant differences among experimental groups (*p* < 0.05), as determined by one-way ANOVA followed by Tukey’s multiple comparison test. Groups sharing at least one lowercase letter are not significantly different.

**Figure 6:**
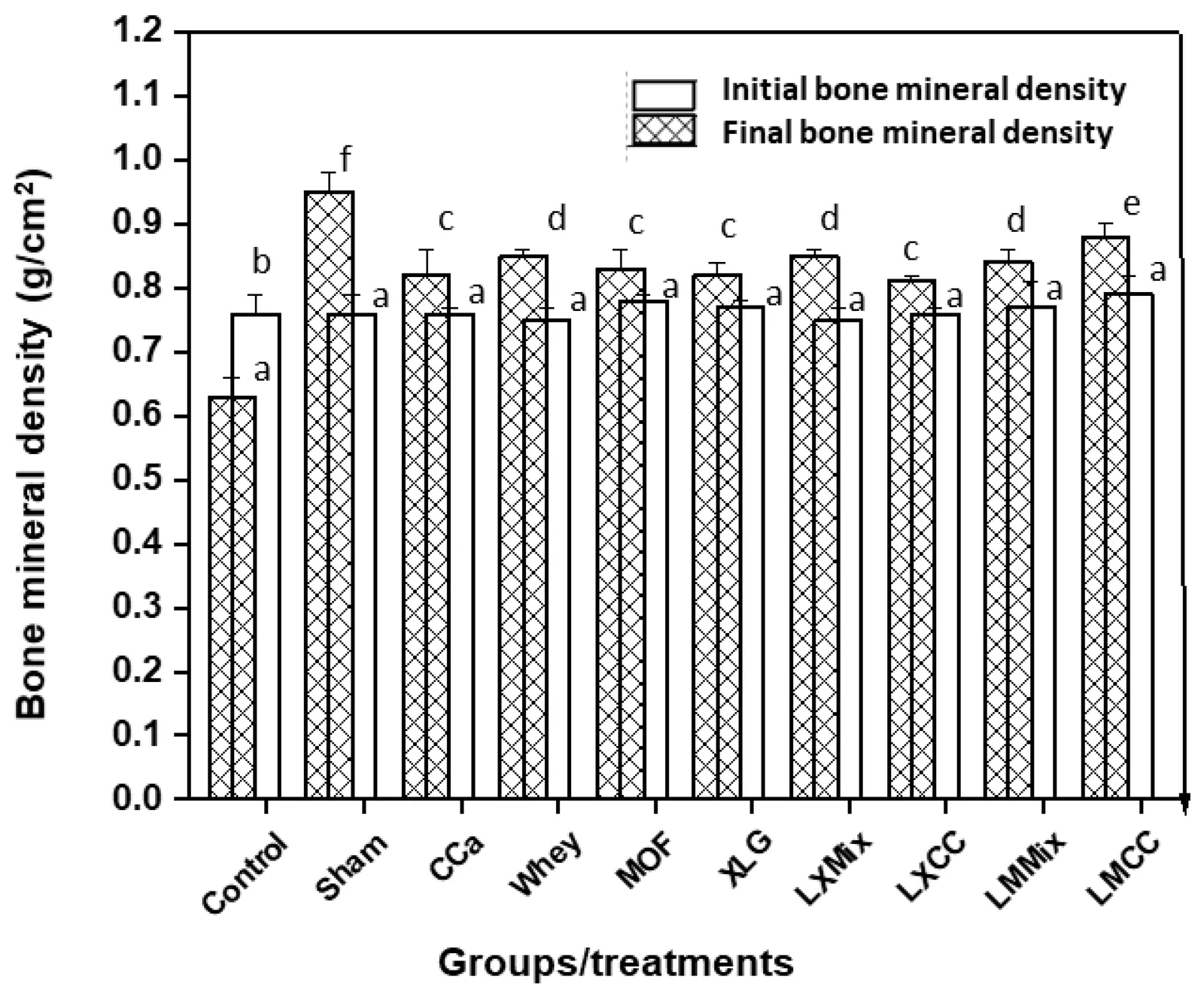
Femoral bone mineral density measured at the beginning and end of the experimental period in the different treatment groups. Data are expressed as mean ± SD (*n* = 8 per group). Different letters indicate significant between-group differences within the corresponding measurement time (*p* < 0.05).

**Figure 7:**
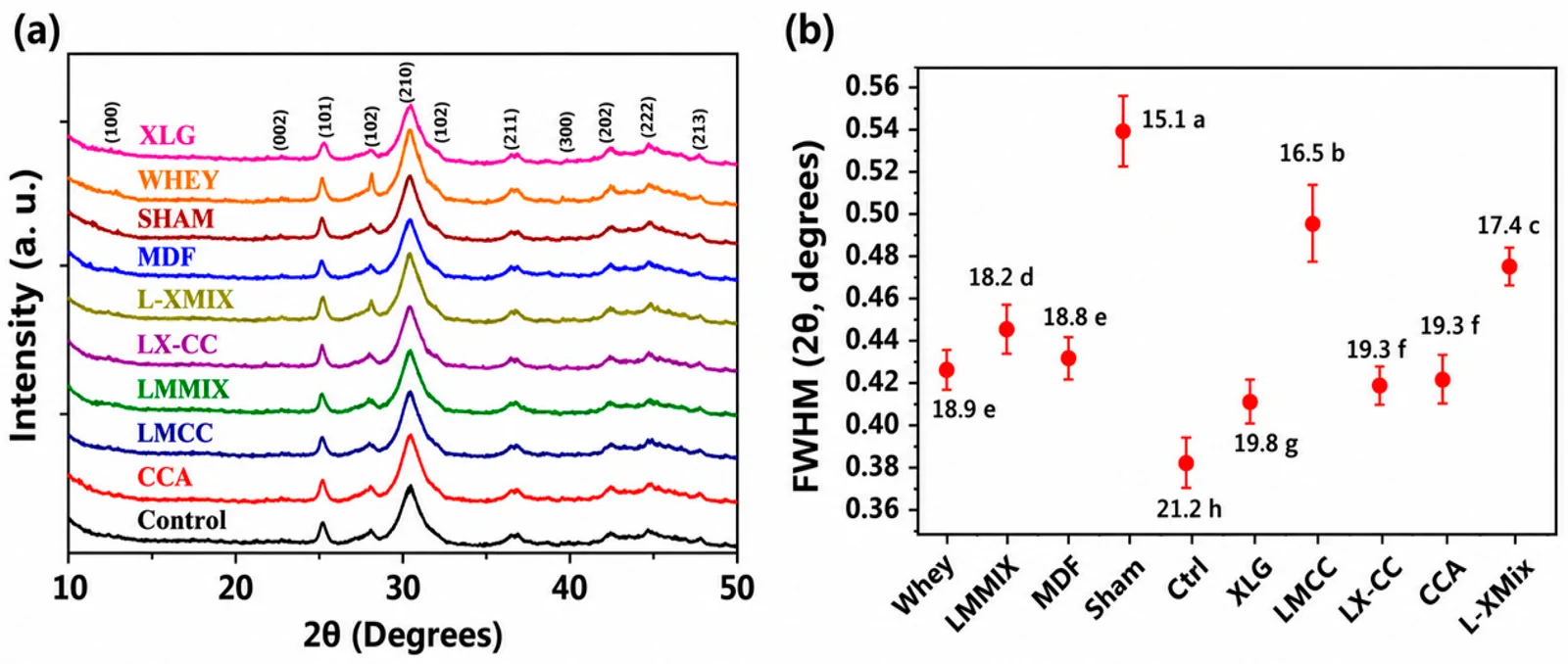
(**a**) X-ray diffraction patterns of femoral bones from experimental groups. (**b**) Full width at half maximum (FWHM) of the hydroxyapatite (002) reflection. Crystal size values (nm), calculated using the Scherrer equation, are indicated above each experimental group. Data are expressed as mean ± SD (*n* = 8 per group). Different letters indicate significant differences among groups (*p* < 0.05).

**Table 1:** Composition and calculated nutritional content of the experimental diets.

	Groups	Sham	Ctrl	CCa	Whey	MOF	XLG	LMMix	LMCC	LXMix	LXCC
Ingredients											
Corn starch (g)		620	620	620	597	620	620	616.5	616.5	616.4	616.5
Sucrose (g)		100	100	100	100	100	100	100	100	100	100
Casein ^a^ (g)		140	140	140	0	140	140	138	137.5	138	140
Soybean oil (g)		40	40	40	37.9	40	40	39.3	39	39.3	39
Fiber ^b^ (g)		50	50	50	50	-	-	49.9	49.8	49.9	49.8
Mineral mix ^c^ (g)		35	35	35	35	35	35	35	35	35	35
Vitamin mix ^d^ (g)		10	10	10	10	10	10	10	10	10	10
L-Cysteine (g)		1.8	1.8	1.8	1.8	1.8	1.8	1.8	1.8	1.8	1.8
Choline bitartrate (g)		2.5	2.5	2.5	2.5	2.5	2.5	2.5	2.5	2.5	2.5
CaCO_3_ ^e^ (g)		2.23	2.23	-	11.2	-	12.3	2.4	2.4	2.4	3.7
Calcium citrate (g)		-	-	3.8	-	-	-	-	-	-	-
Whey (g)		-	-	-	160	-	-	2.83	2.83	0.283	2.83
Mucilage (g)		-	-	-	-	50	-	0.088	0.088	-	-
Xyloglucan (g)		-	-	-	-	-	50.7	-	-	0.088	0.088
Carbohydrates (% Cal)		77.80	77.80	77.80	77.70	77.80	77.80	77.80	77.80	77.80	77.50
Proteins (% Cal)		12.46	12.46	12.46	12.60	12.46	12.46	12.47	12.42	12.43	12.77
Lipids (% Cal)		9.72	9.72	9.72	9.63	9.72	9.72	9.70	9.75	9.67	9.69
Calculated calcium content (mg/kg)		5001	5001	5013	5036	5074	4999	4992	4992	4990	4991
Energy (kcal/kg)		3701	3701	3701	3684	3701	3701	3685	3700	3696	3714

Ctrl, ovariectomized rats fed AIN-93M diet; CCa, calcium citrate; LMMix, whey–mucilage mixture; LMCC, whey–mucilage nanospheres; LXMix, whey–xyloglucan mixture; LXCC, whey–xyloglucan nanospheres; MOF, *Opuntia ficus-indica* mucilage; XLG, *Tamarindus indica* xyloglucan; Whey, cow’s milk whey; Sham, sham-operated control group. Fiber corresponded to α-cellulose and was replaced by the respective polysaccharide in MOF and XLG diets. ^a^ Casein Sigma Chemical, Inc., St. Louis, MO, USA, C-7078; ^b^ α-Cell Fiber Solft Zolca MPbiomedical, Santa Ana, CA, USA; ^c^ Mineral mix without calcium (AIN-93-MX, Harlan Inc., Indianapolis, IN, USA, TD 04374) [30]; ^d^ Vitamin mix (AIN-93-VX, Harlan Inc., Indianapolis, IN, USA, TD 94047) [30]; ^e^ Calcium carbonate (Merck 2066, Darmstadt, Germany) was used as a calcium source in the diets indicated in the table.

**Table 2:** Body weight, estimated food intake, and estimated calcium intake in experimental rats.

Group	Initial Body Weight (g)	Final Body Weight (g)	Food Intake (g/rat/Day)	Estimated Ca Intake (mg/rat/Day)
Sham	250.00 ± 18.67 ^b^	299.86 ± 27.76 ^b^	26.22 ± 1.10 ^ab^	131.09 ± 5.50 ^ab^
Ctrl	292.75 ± 28.70 ^ab^	308.75 ± 38.88 ^ab^	30.24 ± 1.74 ^bc^	151.19 ± 8.70 ^bc^
CCa	336.20 ± 38.36 ^ab^	350.60 ± 44.54 ^ab^	23.87 ± 0.88 ^a^	119.37 ± 4.40 ^a^
Whey	291.00 ± 22.18 ^ab^	313.50 ± 31.14 ^ab^	30.43 ± 0.62 ^c^	152.15 ± 3.10 ^c^
XLG	324.25 ± 17.06 ^a^	336.75 ± 33.39 ^ab^	30.31 ± 1.05 ^c^	151.56 ± 5.25 ^c^
MOF	303.20 ± 29.11 ^ab^	359.00 ± 26.45 ^ab^	24.36 ± 0.38 ^a^	121.80 ± 1.90 ^a^
LXCC	310.00 ± 42.61 ^ab^	360.75 ± 46.14 ^ab^	30.23 ± 0.93 ^c^	151.17 ± 4.65 ^c^
LXMix	357.25 ± 25.13 ^a^	377.25 ± 33.49 ^ab^	30.39 ± 0.19 ^c^	151.95 ± 0.95 ^c^
LMCC	362.80 ± 17.80 ^a^	416.00 ± 23.71 ^a^	30.29 ± 0.25 ^c^	151.46 ± 1.25 ^c^
LMMix	364.00 ± 18.20 ^a^	350.25 ± 27.10 ^ab^	30.43 ±0.18 ^c^	152.15 ± 0.90 ^c^

Values are expressed as mean ± SD (*n* = 8 rats per group). Food intake is expressed as g/rat/day. Calcium intake was estimated from food intake using the formulated dietary calcium concentration of 5 mg/g. Initial and final body weights were analyzed separately using Welch’s ANOVA with Holm-adjusted pairwise comparisons; food intake and estimated calcium intake were analyzed using one-way ANOVA followed by Tukey’s test. Different superscript letters within the same column indicate significant differences (*p* < 0.05); groups sharing at least one letter are not significantly different.

**Table 3:** Morphometric and biomechanical properties of femurs from ovariectomized rats receiving the experimental treatments.

Groups	Dimensional Properties				Biomechanical Properties	
	Length	Weight	Width	Thickness	Fmax	Pmax
	(cm)	(g)	(mm)	(mm)	(N)	(N)
Ctrl	3.42 ± 0.14 ^a^	0.80 ± 0.07 ^a^	3.69 ± 0.20 ^a^	2.93 ± 0.06 ^a^	483.70 ± 16.5 ^a^	73.40 ± 6.8 ^a^
CCa	3.51 ± 0.16 ^a^	0.90 ± 0.12 ^a^	3.79 ± 0.14 ^a^	3.69 ± 0.20 ^a^	526.80 ±12.3 ^b^	96.86 ± 8.3 ^c^
LMCC	3.66 ± 0.13 ^a^	0.95 ± 0.10 ^a^	3.78 ± 0.19 ^a^	3.10 ± 0.07 ^a^	583.10 ±10.8 ^e^	132.30 ± 12.8 ^f^
LMMix	3.69 ± 0.18 ^a^	0.94 ± 0.04 ^a^	3.95 ± 0.20 ^a^	3.20 ± 0.04 ^a^	529.10 ± 9.3 ^b^	82.10 ± 2.1 ^b^
LXCC	3.71 ± 0.15 ^a^	0.87 ± 0.03 ^a^	3.83 ± 0.10 ^a^	2.99 ± 0.16 ^a^	520.80 ± 10.2 ^b^	109.90 ± 2.9 ^d^
LXMix	3.58 ± 0.12 ^a^	0.93 ± 0.09 ^a^	3.76 ± 0.04 ^a^	2.97 ± 0.02 ^a^	519.60 ± 6.5 ^b^	87.52 ± 1.4 ^b^
MOF	3.68 ± 0.14 ^a^	0.96 ± 0.06 ^a^	3.73 ± 0.10 ^a^	3.12 ± 0.14 ^a^	566.20 ±17.1 ^d^	118.91 ± 1.4 ^e^
Sham	3.65 ± 0.11 ^a^	0.85 ± 0.04 ^a^	3.71 ± 0.17 ^a^	2.97 ± 0.07 ^a^	556.80 ±10.1 ^d^	116.40 ± 8.8 ^e^
Whey	3.58 ± 0.16 ^a^	0.82 ± 0.04 ^a^	3.77 ± 0.18 ^a^	2.96 ± 0.10 ^a^	537.20 ± 7.2 ^c^	98.91 ± 3.7 ^c^
XLG	3.43 ± 0.18 ^a^	0.89 ± 0.06 ^a^	3.80 ± 0.15 ^a^	3.14 ± 0.08 ^a^	519.30 ± 8.50 ^b^	86.26 ± 2.9 ^b^

Values are expressed as mean ± SD (*n* = 8). Different superscript letters within the same column indicate significant differences among groups (*p* ≤ 0.05) according to one-way ANOVA followed by Tukey’s multiple comparison test. Fmax, maximum failure load obtained during compression testing; Pmax, maximum failure load obtained during the three-point bending test. Ctrl, ovariectomized rats fed AIN-93M diet; CCa, ovariectomized rats supplemented with calcium citrate; LMCC, whey–*Opuntia ficus-indica* mucilage nanospheres (coacervate); LMMix, whey–*Opuntia ficus-indica* mucilage mixture; LXCC, whey–*Tamarindus indica* xyloglucan nanospheres (coacervate); LXMix, whey–*Tamarindus indica* xyloglucan mixture; MOF, *Opuntia ficus-indica* mucilage; XLG, *Tamarindus indica* xyloglucan; Whey, cow’s milk whey; Sham, sham-operated non-ovariectomized control group.

## Data Availability

The datasets generated and analyzed during the current study are available from the corresponding author upon reasonable request. The data are not deposited in a public repository.

## Abbreviations

The following abbreviations are used in this manuscript:

Abbreviation	Definition
AIN-93M	American Institute of Nutrition-93 Maintenance Diet
ANOVA	Analysis of Variance
BMD	Bone Mineral Density
Ca	Calcium
CaCO_3_	Calcium Carbonate
Ca/P	Calcium-to-Phosphorus Ratio
CCa	Calcium Citrate Group
CO_2_	Carbon Dioxide
Ctrl	Ovariectomized Control Group
Ct.Th	Cortical Thickness
ELISA	Enzyme-Linked Immunosorbent Assay
CI	Confidence Interval
BF-STEM	Bright Field Scanning Transmission Electron Microscopy
Fmax	Maximum Compressive Failure Load
FWHM	Full Width at Half Maximum
HA	Hydroxyapatite
ICP-OES	Inductively Coupled Plasma Optical Emission Spectrometry
LMCC	Whey–*Opuntia ficus-indica* Mucilage Nanospheres (Complex Coacervate)
LMMix	Whey–*Opuntia ficus-indica* Mucilage Physical Mixture
LXCC	Whey–*Tamarindus indica* Xyloglucan Nanospheres (Complex Coacervate)
LXMix	Whey–*Tamarindus indica* Xyloglucan Physical Mixture
MOF	*Opuntia ficus-indica* Mucilage
NOM	Mexican Official Standard (Norma Oficial Mexicana)
OVX	Ovariectomized
P	Phosphorus
PDI	Polydispersity Index
Pmax	Maximum Load during Three-Point Bending Test
RANKL	Receptor Activator of Nuclear Factor κB Ligand
SCFAs	Short-Chain Fatty Acids
SD	Standard Deviation
SEM	Scanning Electron Microscopy
SE(UL)	Secondary Electron Imaging
Tb.Sp	Trabecular Separation
Tb.Th	Trabecular Thickness
XLG	*Tamarindus indica* Xyloglucan
XRD	X-ray Diffraction
ζ-potential	Zeta Potential
